# Expanding the Allelic and Clinical Heterogeneity of Movement Disorders Linked to Defects of Mitochondrial Adenosine Triphosphate Synthase

**DOI:** 10.1002/mds.30209

**Published:** 2025-04-25

**Authors:** Philip Harrer, Magdalena Krygier, Martin Krenn, Volker Kittke, Martin Danis, Georgi Krastev, Alice Saparov, Virginie Pichon, Marlène Malbos, Clarisse Scherer, Ivana Dzinovic, Matej Skorvanek, Robert Kopajtich, Holger Prokisch, Sara Silvaieh, Anna Grisold, Maria Mazurkiewicz‐Bełdzińska, Jean‐Madeleine de Sainte Agathe, Juliane Winkelmann, Jan Necpal, Robert Jech, Michael Zech

**Affiliations:** ^1^ Institute of Human Genetics, School of Medicine and Health, Technical University of Munich Munich Germany; ^2^ Institute of Neurogenomics, Helmholtz Zentrum München Munich Germany; ^3^ Department of Developmental Neurology Medical University of Gdansk Gdansk Poland; ^4^ Department of Neurology Medical University of Vienna Vienna Austria; ^5^ Comprehensive Center for Clinical Neurosciences & Mental Health, Medical University of Vienna Vienna Austria; ^6^ Neurological Clinic of Faculty Hospital Trnava and Slovak Health University Bratislava Bratislava Slovakia; ^7^ Institute for Advanced Study, Technical University of Munich Garching Germany; ^8^ CRMR Neurogenetique, Service de Neurologie, Centre Hospitalier, Universitaire d’Angers Angers France; ^9^ CRMRs “Anomalies du Développement et syndromes malformatifs” et “Déficiences Intellectuelles de causes rares,” FHU‐TRANSLAD, CHU Dijon Bourgogne Dijon France; ^10^ Laboratoire de Génomique Médicale, UF Innovation en diagnostic génomique des maladies rares, CHU Dijon Bourgogne Dijon France; ^11^ Department of Neurology P.J. Safarik University Kosice Slovakia; ^12^ Department of Neurology University Hospital of L. Pasteur Kosice Slovakia; ^13^ Department of Medical Genetics Sorbonne Université, AP‐HP Sorbonne Université Paris France; ^14^ Laboratoire de Biologie Médicale Multi‐Site SeqOIA, Sorbonne Université Paris France; ^15^ DZPG (German Center for Mental Health) Munich Germany; ^16^ Munich Cluster for Systems Neurology, SyNergy Munich Germany; ^17^ Department of Neurology Zvolen Hospital Zvolen Slovakia; ^18^ Parkinsonism and Movement Disorders Treatment Center, Zvolen Hospital Zvolen Slovakia; ^19^ Department of Neurology and Center of Clinical Neuroscience First Faculty of Medicine Charles University and General University Hospital in Prague Prague Czech Republic

**Keywords:** ATP synthase, dystonia, spasticity, cerebral palsy, mitochondrial disease, dominant variant, ATP5F1A, ATP5F1B

## Abstract

**Background:**

Defects of mitochondrial ATP synthase (ATPase) represent an emerging, yet incompletely understood group of neurodevelopmental diseases with abnormal movements.

**Objective:**

The aim of this study was to redefine the phenotypic and mutational spectrum of movement disorders linked to the ATPase subunit‐encoding genes *ATP5F1A* and *ATP5F1B*.

**Methods:**

We recruited regionally distant patients who had been genome or exome sequenced. Fibroblast cultures from two patients were established to perform RNA sequencing, immunoblotting, mass spectrometry–based high‐throughput quantitative proteomics, and ATPase activity assays. In silico three‐dimensional missense variant modeling was performed.

**Results:**

We identified a patient with developmental delay, myoclonic dystonia, and spasticity who carried a heterozygous frameshift c.1404del (p.Glu469Serfs*3) variant in *ATP5F1A*. The patient's cells exhibited significant reductions in *ATP5F1A* mRNA, underexpression of the α‐subunit of ATPase in association with other aberrantly expressed ATPase components, and compromised ATPase activity. In addition, a novel deleterious heterozygous *ATP5F1A* missense c.1252G>A (p.Gly418Arg) variant was discovered, shared by three patients from two families with hereditary spastic paraplegia (HSP). This variant mapped to a functionally important intersubunit communication site. A third heterozygous variant, c.1074+1G>T, affected a canonical donor splice site of *ATP5F1B* and resulted in exon skipping with significantly diminished *ATP5F1B* mRNA levels, as well as impaired ATPase activity. The associated phenotype consisted of cerebral palsy (CP) with prominent generalized dystonia.

**Conclusions:**

Our data confirm and expand the role of dominant *ATP5F1A* and *ATP5F1B* variants in neurodevelopmental movement disorders. *ATP5F1A*/*ATP5F1B*‐related ATPase diseases should be considered as a cause of dystonia, HSP, and CP. © 2025 The Author(s). *Movement Disorders* published by Wiley Periodicals LLC on behalf of International Parkinson and Movement Disorder Society.

Adenosine triphosphate (ATP) production in the nervous system is tightly controlled and vital for neuronal integrity.^1^ Central to this process is mitochondrial ATP synthase (ATPase), a macromolecular complex conserved across most eukaryotic lineages.^2^ ATPase encompasses 17 different structural subunits, organized into a membrane‐embedded F_O_ sector and the membrane‐extrinsic F_1_ domain.^2^, ^3^ There are only a modest number of reports linking variants in ATPase‐subunit genes to heritable human disorders,^3^, ^4^, ^5^ likely because of the essential function of the complex for cellular bioenergetic requirements^2^ and the resulting mutation constraint.^6^ Elsewhere, we have identified a group of three unrelated patients harboring variants in *ATP5F1A*, which codes for the α‐subunit of ATPase, and also have begun to show that the associated clinical outcomes are heterogeneous, ranging from transient neurodevelopmental impairment to chronic movement disorder syndromes with dystonia and spasticity.^7^ Although rare, these ATPase‐related phenotypes reinforced a connection between mitochondrial defects and movement disorders, and highlighted new opportunities to study clinically relevant mechanisms of ATPase dysfunction.^8^ In addition, our group contributed to the description of two families affected by isolated dystonia who carried candidate variants in the ATPase β‐subunit gene *ATP5F1B*, but this gene–disease relationship awaits further confirmation.^9^

As interaction partners in the F_1_ part of ATPase, the α‐ and β‐subunits directly catalyze the synthesis of ATP.^2^ Unsurprisingly, both *ATP5F1A* and *ATP5F1B* are variant‐intolerant genes, statistically depleted of missense and loss‐of‐function (LoF) changes (gnomAD‐v4.1.0: missense *Z* scores > 3; LoF observed/expected upper bound fraction [LOEUF] scores < 0.6).^6^ Disease‐related *ATP5F1A*/*ATP5F1B* alleles implicated in movement disorders to date were monoallelic missense variants, variably causing compromised ATPase activity and/or reductions in subunit expression levels.^7^, ^9^, ^10^, ^11^ In extremely rare cases, *ATP5F1A* missense variants have also been detected in an autosomal‐recessive disease context, implying the existence of allelic heterogeneity.^12^, ^13^ Heterozygous LoF variants in *ATP5F1A* or *ATP5F1B* have not yet been found in published patients.

In this study, we combined whole‐genome/exome sequencing (WGS/WES), international data sharing, RNA sequencing (RNAseq), immunoblotting, high‐throughput proteomics, enzymatic studies, and in silico predictions to expand the genotypical and clinical spectrum of ATPase‐related disorders caused by variants in *ATP5F1A* and *ATP5F1B*. We now provide evidence that (1) heterozygous LoF mutations can be causative for *ATP5F1A*/*ATP5F1B*‐associated dystonia; (2) a novel *ATP5F1A* missense substitution, occurring recurrently in independent subjects, leads to lower‐limb spasticity mimicking other classical genetic forms of hereditary spastic paraplegia (HSP); and (3) a canonical splice‐site alteration in *ATP5F1B* is linked to the diagnosis of dystonic cerebral palsy (CP), differently from previously reported missense variant‐associated isolated dystonia.

## Subjects and Methods

### Participants

**FIG. 1:**
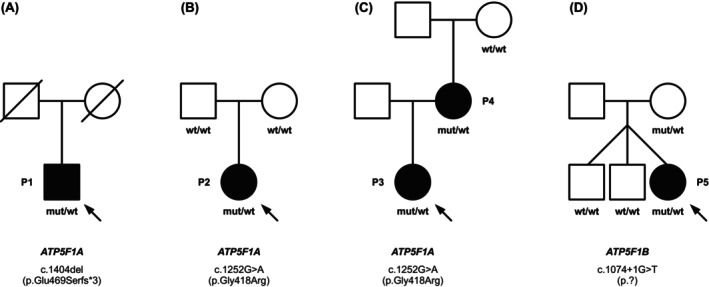
Pedigree drawings for families with heterozygous variants in *ATP5F1A* or *ATP5F1B*. (**A**) Family A with patient 1 (P1). (**B**) Family B with P2. (**C**) Family C with P3 (daughter) and P4 (mother). (**D**) Family D with P5. Closed symbols indicate the individuals affected by movement disorders; mut/wt denotes the reported *ATP5F1A*/*ATP5F1B* variants in the heterozygous state; and wt/wt denotes a homozygous wild‐type allele. Index patients are marked with arrows. The variant in P2 was confirmed to be de novo by targeted Sanger testing of the parents.

Previously unreported patients with variants in *ATP5F1A* (patient 1 [P1]–P4, families A–C) or *ATP5F1B* (P5, family D) and healthy relatives were evaluated for this study (Fig. 1, Table S1). Affected subjects included four females and one male, and ranged from 14 to 61 years of age (Table S1). The families were recruited via collaborations enabled by GeneMatcher^14^ and direct communications from neurological and neuropediatric specialty centers in four countries (Austria, France, Poland, and Slovakia). All participants or their parents/caretakers provided informed consent for research and/or diagnostic studies according to the Declaration of Helsinki and institutional review board requirements at their care centers. The clinical enrollment procedures involved retrospective review of medical records, history taking, and prospective examinations.

### Genomic Sequencing

Genomic DNA was extracted from peripheral blood and analyzed by WGS (P1, family A; P5, family D) or WES (P2, family B; P3/P4, family C). Details of the WGS and WES methods have been described elsewhere.^15^, ^16^, ^17^ Paired‐end 150‐bp (WGS) or 100‐bp (WES) sequencing was performed on Illumina machines, and the output was processed with published pipelines according to accredited standards.^15^, ^17^ Annotated variants were filtered and classified following recommended guidelines.^18^ Sequencing data from P1, P2, and P5 were evaluated within a large dataset of rare movement disorders of suspected Mendelian inheritance on the Munich WES/WGS analysis platform (https://github.com/mri-ihg/EVAdb; Munich, Germany), facilitating the discovery of shared genetic etiologies.^7^ WES variant profiles from P3/P4 were assessed with an established in‐house protocol, integrating queries of ClinVar‐listed likely pathogenic/pathogenic variants.^17^ *ATP5F1A*/*ATP5F1B* variants were Sanger validated and tested in samples of relatives, whenever available. The variants were classified according to criteria of the American College of Medical Genetics and Genomics (ACMG).^18^

### Fibroblast Culture

Primary fibroblast lines were established in‐house from skin biopsy specimens from P1 (family A) and P5 (family D), as previously reported.^7^

### RNAseq

RNA isolated from cultured fibroblasts from P1 and P5 was subjected to RNAseq using the TruSeq Stranded mRNA Sample Prep LS Protocol following the manufacturer's instructions.^7^ Quality control was done as described, and libraries were processed on a NovaSeq6000. The resulting data were studied using an in‐house–established pipeline specifically designed to monitor aberrant expression.^19^ As controls, a large set of >750 fibroblasts from individuals with unrelated monogenic conditions was used.^20^ An *ATP5F1B* mRNA splicing defect was visualized with the Integrative Genomics Viewer (https://igv.org/).

### Immunoblotting

Total protein extracts were prepared from fibroblast lysates for P1, P5, and three control subjects.^7^ Immunoblot membranes were probed with anti‐ATP5F1A (459240, 1:2000; Invitrogen), anti‐ATP5F1B (ab14730, 1:2000; Abcam), anti‐ATP5PD (ab110275, 1:1000; Abcam), anti‐ATP5PO (ab110276, 1:1000; Abcam), anti‐ATP5F1C (NBP2‐15525, 1:1000; Novus), and anti‐ATP5F1D (PA5‐21361, 1:1000; Invitrogen) as primary antibodies. The blots were then incubated with the appropriate secondary antibodies anti‐GAPDH (2118, 1:10,000; Cell Signaling), mouse anti‐β‐Tubulin (66240‐1‐Ig, 1:10,000; Proteintech), or rabbit anti‐β‐Tubulin (11‐13002, 1:10,000; Abeomics). Results were quantified with Bio‐Rad Image Lab. Three biological replicates were analyzed for each subject. Statistical significance was determined by Student's *t* test using R.

### Tandem Mass Tag–Labeled Quantitative Proteomics

Quantitative tandem mass tag proteomics analysis was conducted at the BayBioMS core facility of Technical University of Munich (Freising, Germany) on a Fusion Lumos Tribrid mass spectrometer (Thermo Scientific), as reported in our recent work.^21^ In brief, cell pellets derived from cultured primary fibroblasts (0.5 million cells per pellet) were lysed under denaturing conditions in a buffer containing urea and quantified using the BCA Protein Assay Kit (Thermo Fisher Scientific, Waltham, MA, USA). Protein extract (15 μg) was reduced and alkylated. Tryptic digestion was done by using Trypsin Gold (Promega, Madison, WI, USA). Following mass‐spectrometry measurements, the MaxQuant platform^22^ was used for peptide identification, and protein groups were determined. Standardized protein expression outlier discovery was performed by using an in‐house–established bioinformatics processing pipeline (https://github.com/gagneurlab/py_outrider),^21^ which provided for each sample a catalog of aberrantly expressed proteins with corresponding multiple testing‐corrected *P* values and fold‐change information. The overall proteomics test cohort comprised 213 proteomes (100 from patients with dystonia, 113 from independent research subjects with rare disorders).^21^ We focused on underexpressed proteins with adjusted *P* < 0.05.

### ATPase Enzymatic Testing

The ATPase Activity Microplate Assay Kit (ab109716; Abcam) was used to determine the activity of ATPase in cultured fibroblasts from P1, P5, and two age‐ and sex‐matched control subjects, in accordance with the manufacturer's specifications. Three biological replicates with three technical replicates per biological replicate were analyzed for each subject. To determine statistical significance, we used the mean of the three technical replicates for each biological replicate. Significance was calculated by Student's *t* test using R.

### In Silico Analysis

An array of computational tools was used to predict the consequence of identified missense and splice‐site variants in *ATP5F1A*/*ATP5F1B*, including CADD,^23^ REVEL,^24^ AlphaMissense,^25^ and SpliceAI.^26^ A model of ATPase was created using the Protein Data Bank (PDB) template 8H9I, and the three‐dimensional location of a missense variant in ATP5F1A was analyzed in relation to functional protein structures. In addition, the missense variant was modeled with SWISS‐MODEL^27^ using the PDB template 6ZPO and visualized using Mol* Viewer.^28^

## Results

### ATP5F1A c.1404del (p.Glu469Serfs*3)

**FIG. 2:**
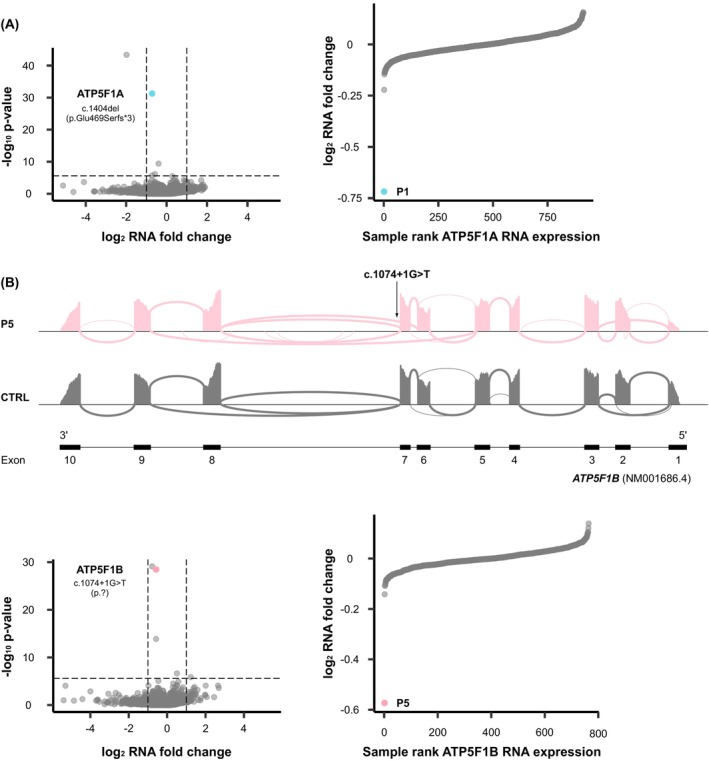
RNA sequencing (RNAseq) studies showing reduced expression of *ATP5F1A* and *ATP5F1B*. (**A**) Significantly reduced *ATP5F1A* mRNA levels in patient 1 (P1; family A) affected by the heterozygous frameshift variant c.1404del (p.Glu469Serfs*3). Transcriptomics volcano and sample rank plots are shown. In the volcano plot, the underexpression outlier *ATP5F1A* is indicated in blue; the horizontal line represents a *P* value of 2.5 × 10^−6^ (corrected *P* value for 20,000 hypotheses corresponding to the number of theoretically identifiable gene‐derived RNAs), and the vertical line represents log2fold changes of ±1. In comparison with all in‐house RNAseq control samples (n > 750), P1 had the lowest amounts of *ATP5F1A* mRNA (blue data point in the sample rank plot). (**B**) Missplicing and significantly reduced *ATP5F1B* mRNA levels in P5 (family D) affected by the heterozygous splice‐site variant c.1074+1G>T. Transcriptomics sashimi, volcano, and sample rank plots are shown. Sashimi plots for P5 and a representative control subject demonstrate the occurrence of exon skipping (exon 7, exons 6 plus 7) as a result of the patient‐specific G>T substitution at the donor splice site of exon 7. The number of reads spanning each junction is highlighted by the size of the sashimi‐plot curve. In the volcano plot, the underexpression outlier *ATP5F1B* is indicated in pink; the horizontal line represents a *P* value of 2.5 × 10^−6^ (corrected *P* value for 20,000 hypotheses corresponding to the number of theoretically identifiable gene‐derived RNAs), and the vertical line represents log2fold changes of ±1. In comparison with all in‐house RNAseq control samples (n > 750), P5 had the lowest amounts of *ATP5F1B* mRNA (pink data point in the sample rank plot). [Color figure can be viewed at wileyonlinelibrary.com]

**FIG. 3:**
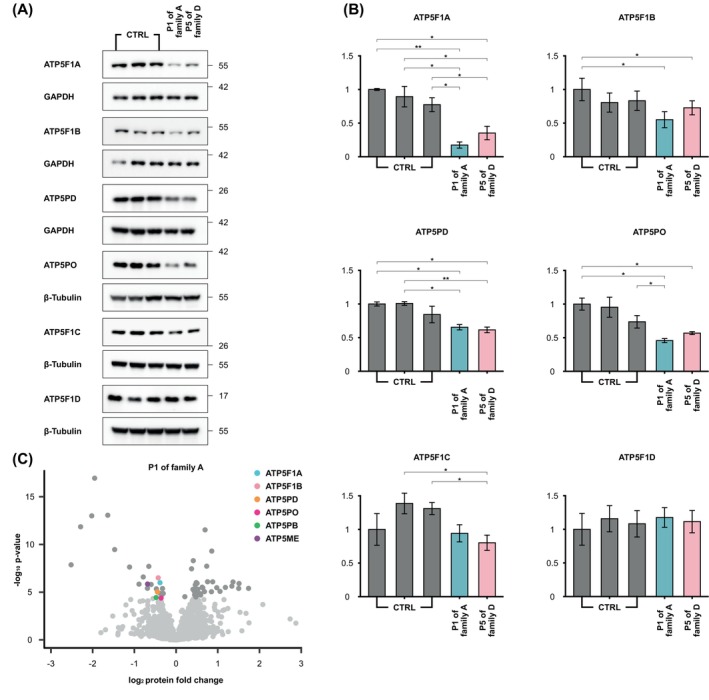
Effect of variants in *ATP5F1A* and *ATP5F1B* on ATP synthase subunit expression. Immunoblot analysis of ATP5F1A, ATP5F1B, ATP5PD, ATP5PO, ATP5F1C, and ATP5F1D was performed in fibroblast lysates from three unrelated control subjects (CTRL), patient 1 (P1; family A) affected by the heterozygous *ATP5F1A* frameshift variant c.1404del (p.Glu469Serfs*3), and P5 (family D) affected by the heterozygous *ATP5F1B* splice‐site variant c.1074+1G>T. Three biological replicates were analyzed, and representative images are shown in (**A**). In the quantifications (**B**), the values shown represent means ± standard deviations. Significances were assessed by Student's *t* test; *P ≤ 0.05, **P ≤ 0.01. (**C**) Proteomics volcano plot for P1 (family A) is shown. Darker gray dots represent proteins whose expression was significantly altered (adjusted *P* value threshold of 0.05), and lighter gray dots represent proteins whose expression was not significantly altered (adjusted *P* > 0.05). The significant underexpression outliers ATP5F1A, ATP5F1B, ATP5PD, ATP5PO, ATP5PB, and ATP5ME are highlighted in different colors, as indicated. [Color figure can be viewed at wileyonlinelibrary.com]

**FIG. 4:**
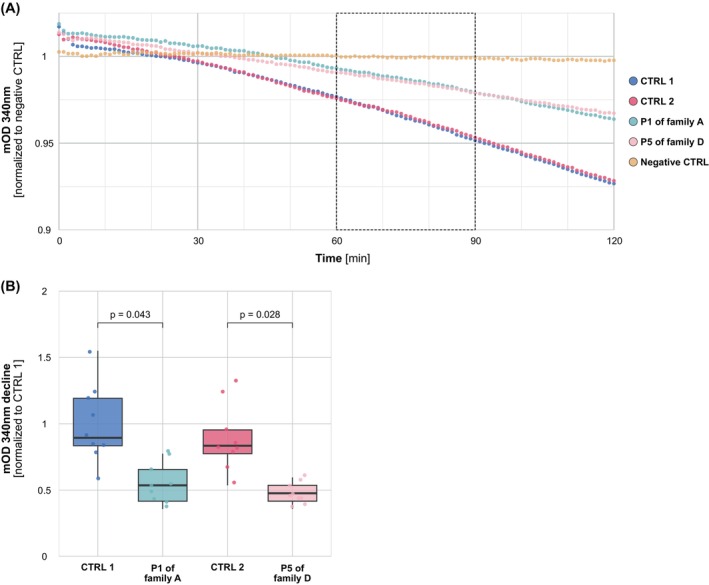
ATP synthase activity studies using fibroblasts from patients with variants in *ATP5F1A* and *ATP5F1B*. (**A**) Representation of ATPase activity, evaluated by the ATP synthase Activity Microplate Assay Kit (Abcam), in the fibroblasts from two age‐ and sex‐matched control subjects (CTRL 1/2), patient 1 (P1; family A) affected by the heterozygous *ATP5F1A* frameshift variant c.1404del (p.Glu469Serfs*3), and P5 (family D) affected by the heterozygous *ATP5F1B* splice‐site variant c.1074+1G>T. Data represent three biological replicates with three technical replicates per biological replicate. Boxplots (**B**) represent the median, first quartile, and third quartile, and whiskers extend to a maximum of 1.5× interquartile range. Significances were assessed by Student's *t* test. [Color figure can be viewed at wileyonlinelibrary.com]

In P1 (family A; Fig. 1, Table S1), WGS uncovered a heterozygous c.1404del variant in *ATP5F1A* (NM_004046.6), predicted to result in a shift of the open reading frame and insertion of a premature stop codon in exon 10 (of 12 total exons; p.Glu469Serfs*3). Relatives were unavailable for segregation analysis. The variant was not reported in either gnomAD (v4.1.0) or our in‐house databases (>30,000 WES/WGS datasets). We used RNAseq in fibroblasts to assess the impact of c.1404del on *ATP5F1A* mRNA abundance. As shown in Figure 2A, *ATP5F1A* was a significant underexpression outlier in P1's transcriptome (fold change = 0.59), and this patient exhibited the lowest *ATP5F1A‐*mRNA expression among in‐house–sequenced fibroblast samples. Immunoblotting indicated that ATP5F1A protein was significantly downregulated in P1 when compared with normal expression (Fig. 3A,B); this effect was accompanied by a trend toward reduced levels of other examined ATPase subunits (ATP5F1B, ATP5PD, and ATP5PO; Fig. 3A,B) on immunoblotting, consistent with the prior observation that nonassembled ATPase components are subject to proteolytic degradation.^12^, ^29^ To verify and expand these observations, we further undertook unbiased proteomic analysis on P1's fibroblasts using a previously described experimental setting based on mass spectrometry.^21^ This profiling demonstrated a remarkably consistent decrease in steady‐state expression levels across several protein subunits of ATPase, suggesting that c.1404del profoundly impacted the integrity of the complex in P1 (Fig. 3C); P1's cells displayed the lowest abundance of ATP5F1A (fold change = 0.77) and five other significantly underexpressed ATPase subunits in comparison with a cohort of 212 individuals with available fibroblast proteomes (ATP5F1B, fold change = 0.74; ATP5PD, fold change = 0.73; ATP5PO, fold change = 0.78; ATP5PB, fold change = 0.72; ATP5ME, fold change = 0.62) (Fig. S1). In addition, enzymatic testing showed markedly decreased total ATPase activity in P1's fibroblasts relative to control cells (Fig. 4A,B). Following ACMG rules,^18^ we classified *ATP5F1A* c.1404del as “likely pathogenic” (Table S1); we applied the PS3 criterion^18^ to reflect the results of our functional studies including mRNA and protein expression profiling, as well as testing of ATPase enzymatic function. P1 was a Slovak man with developmental delay, combined dystonia, and cognitive impairment (Table S1). He presented at 12 months of age with hypotonia and delayed motor milestones. At 3 years of age, he developed limb dystonic posturing; the disease progressed, and at the age of 43 years he had a mixed movement phenotype with generalized dystonia, myoclonus, and a pyramidal syndrome involving predominantly the legs (Video 1). His brain magnetic resonance imaging (MRI) scan was normal, and his family history was negative.

**Video 1:** Patient P1 (family A) at the age of 43 years. Examination shows generalized dystonia involving the limbs, trunk, and craniocervical region, accompanied by a prominent myoclonic component.

### ATP5F1A c.1252G>A (p.Gly418Arg)

**FIG. 5:**
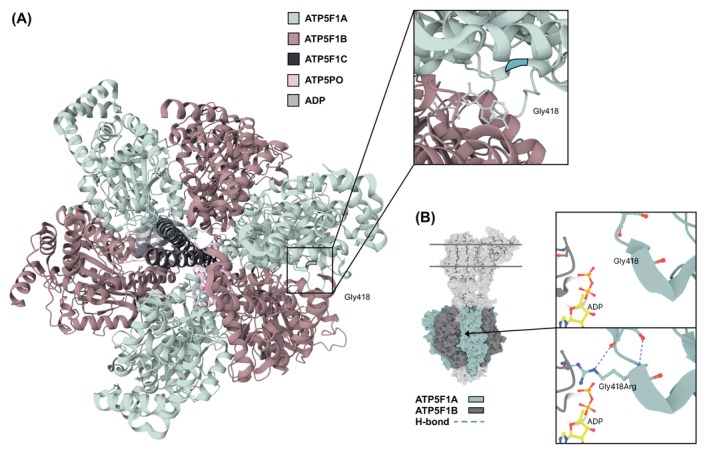
Location and structural modeling of the ATP5F1A missense variant p.Gly418Arg. (**A**) A three‐dimensional representation of the human ATPase F_1_ domain (PDB: 8H9I) is shown, with major subunits highlighted in the indicated colors. The residue Gly418 mutated in families B and C is situated at the α‐β intersubunit interaction space (ATP5F1A‐ATP5F1B). Moreover, Gly418 is in close proximity to the ADP binding site, as highlighted in the magnified view of the sequence containing this amino acid; ADP is shown in stick‐and‐ball representation (gray). (**B**) Model of ATPase and magnified view of the predicted p.Gly418Arg substitution in the α‐β communication space (PDB: 6ZPO). Substitution of the nonpolar Gly418 by a positively charged arginine may perturb functionally important subunit interactions and/or structural features that sustain binding (or nonbinding) behaviors. As illustrated by blue dashed lines, Arg418 may introduce new intermolecular bonding interactions (H‐bonds) involving the α‐ and β‐subunits. [Color figure can be viewed at wileyonlinelibrary.com]

WES detected the heterozygous *ATP5F1A* missense variant c.1252G>A (p.Gly418Arg) in P2 (family B; Fig. 1, Table S1). A de novo origin of the variant was demonstrated by Sanger testing of the parents. The same c.1252G>A substitution was shown by duo WES in the independent mother–daughter pair P3/P4 (family C; Fig. 1, Table S1). Control databases (gnomAD v4.1.0, in‐house collections) did not contain the variant, and conservation analysis showed that the mutated amino acid site was invariant across all examined species (data not shown), suggesting functional importance. In silico predictions of the variant highlighted its likely deleterious nature (Table S1). To assess how the variant could impair ATP5F1A function, we investigated structural and physicochemical properties of Gly418; the residue was positioned in the vicinity of ATP5F1A's β‐subunit–communication interface, indicating that replacement of the nonpolar glycine with a positively charged arginine may alter the ability of ATP5F1A to interact with ATP5F1B (Fig. 5A,B). We classified *ATP5F1A* c.1252G>A as “likely pathogenic”^18^ (Table S1). At the time of last examination, P2 was a 22‐year‐old Austrian woman with spastic gait impairment and without any relevant family history (Table S1). She had motor delay during childhood, and by the age of 14 years she developed slowly progressive spasticity of the legs (Video 2). This led to recurrent falls since adulthood. A diagnosis of HSP was given. Psychometric tests disclosed mild intellectual disability. Brain MRI was normal except for presence of an optic glioma. In family C with familial HSP from France, P3 was a 32‐year‐old woman who exhibited spastic paraparesis and mild neurodevelopmental impairments (Table S1); she had delayed language development, borderline intellectual disability, and autism spectrum disorder. Her 61‐year‐old mother (P4) displayed a phenotype dominated by bilateral lower‐extremity spasticity, but without recognizable developmental comorbidity. Both individuals had unremarkable brain MRI results.

**Video 2:** Patient P2 (family B) at the age of 22 years. Examination shows a bilateral pyramidal syndrome in the lower limbs consistent with a clinical diagnosis of hereditary spastic paraplegia.

### ATP5F1B c.1074+1G>T

A heterozygous c.1074+1G>T variant in *ATP5F1B* (NM_001686.4) was discovered by WGS in P5 (family D; Fig. 1, Table S1). Sanger sequencing demonstrated that P5's healthy mother also carried this variant heterozygously, whereas her two triplet brothers were homozygous for the wild‐type allele. The variant was unobserved in the gnomAD v4.1.0 and in‐house control cohorts. SpliceAI predicted that the variant would weaken the canonical donor splice site of exon 7 (donor loss with high confidence; score of 1.0). RNAseq data from fibroblasts confirmed the presence of a splice defect with skipping of exon 7 and exons 6 plus 7 (Fig. 2B); *ATP5F1B* mRNA levels were significantly decreased (fold change = 0.67; Fig. 2B). The polypeptide encoded by exon 7 included Thr334, the residue mutated in a recently reported family with isolated dystonia.^9^ Immunoblot analysis on P5's fibroblasts showed that the cells contained significantly reduced amounts of the ATPase component ATP5F1A, whereas *ATP5F1B* expression was not consistently altered at the protein level in the examined fibroblasts (Fig. 3A,B). Total ATPase activity was significantly diminished in these fibroblasts (Fig. 4A,B). Given the validated effect of c.1074+1G>T on *ATP5F1B* mRNA splicing with observable impairment of ATPase function, the variant was classified as “likely pathogenic”^18^ (Table S1); we considered the PS3 and PM4 (exon skipping with in‐frame deletions) rules^18^ to upgrade the significance of the variant from uncertain to class 4 evidence. P5 was a Ukrainian girl with motor delay and a diagnosis of CP (Table S1). Family history was noncontributory. Her delivery was complicated by prolonged labor (32 weeks of gestation, APGAR score of 7), but postnatal adaption was normal. At last evaluation, she was 14 years old and able to walk only a few steps with maximum assistance; there were four‐limb dystonic and distal‐arm athetotiform movements, truncal dystonia, and cervical dystonia (Video 3). Brain MRI studies indicated no pathology.

**Video 3:** Patient P5 (family D) at the age of 14 years. Examination shows generalized dystonia with involvement of the limbs, trunk, and neck. The clinical picture with infantile onset and a history of complicated birth led to a diagnosis of dystonic cerebral palsy.

## Discussion

Because ATPase is the major engine to generate ATP for almost all intracellular metabolic pathways, its defects may have multiple detrimental effects on neurophysiology depending on the degree of functional impairment.^3^ A severe decrease in the abundance of ATPase was found in autosomal recessively or mitochondrially inherited subunit deficiencies, typically linked to devastating encephalopathies with early lethality.^12^, ^30^, ^31^ Although a handful of heterozygous single amino acid substitutions in *ATP5F1A* and *ATP5F1B*, encoding the key ATP‐synthesizing subunits of ATPase, have recently been associated with different types of dystonic‐spastic syndromes (for details, see Table S1),^7^, ^9^ the implication of these genes in movement disorders is not well characterized. In this study, we have gathered a set of five patients from four unrelated families with autosomal dominant variants in *ATP5F1A* or *ATP5F1B*, further defining the genetic, molecular, and phenotypic profile of ATPase‐related diseases with dystonia and spasticity.

We describe a heterozygous frameshift variant in *ATP5F1A* resulting in reduced mRNA and ATP5F1A‐protein levels, consistent with haploinsufficiency. The disorder combining features of early‐onset dystonia, pyramidal signs, and neurodevelopmental disability of the individual carrying this alteration resembled the clinical picture of two published patients with missense *ATP5F1A* mutations.^7^ Several lines of evidence support that the lack of normal ATP5F1A expression in individual P1 was disease related. First, decreased amounts of other subunits, including ATP5F1A's F_1_ interaction partners, were seen in P1 on immunoblot analysis, indicative of overall diminished abundance of functional ATPase. Importantly, we were able to significantly replicate this finding by performing proteomic analysis on the patient‐derived primary fibroblast cell line. In this experiment, we observed markedly changed levels of six ATPase protein subunits, confirming that the identified heterozygous *ATP5F1A* frameshift variant deleteriously affected the normal composition of the ATPase complex in P1. Besides ATP5F1A, proteomics also detected significant reduction (adjusted *P* < 0.05) in the expression levels of ATP5F1B, ATP5PD, ATP5PO, ATP5PB, and ATP5ME in this cell line (Fig. 3C, Fig. S1). Notably, partial loss of normal ATPase expression was previously shown to be causative of mitochondrial dysfunction syndromes with neurological disturbances.^32^, ^33^ Published studies demonstrated that biallelic variants in *ATP5F1A* cause considerably more severe phenotypes in affected individuals, whereas the parents of these patients were reported to be neurologically normal.^12^, ^13^ Although the alleles described in this context were expected to result in reduced ATPase amounts, the variants were missense changes^12^, ^13^; no clear‐cut LoF (ie, frameshift, nonsense, or splice site) variants have been reported in the recessive form of *ATP5F1A*‐related disease. In one recessive family, the defect of ATP5F1A was associated with a missense mutation on the paternal allele and an unidentified second variant causing nonexpression of the maternal allele^12^; however, it remained unclear whether the unknown maternal genotype produced underexpression of ATP5F1A protein and/or other ATPase protein subunits in the unaffected mother,^12^ as seen in our individual P1.

Second, P1's cells exhibited an ATPase activity deficit, highlighting that the complex formed in the presence of the heterozygous *ATP5F1A* frameshift variant is not adequately functional. ATPase amount in mitochondria is closely maintained to guarantee the cell's core bioenergetic supply, and failure to sustain controlled functionality of this complex is a well‐known mechanism in many neurodevelopmental and neurodegenerative syndromes.^1^, ^8^ Third, a similar ATP5F1A protein reduction was reported for a pathogenic missense *ATP5F1A* variant causing ATPase‐related neurodevelopmental symptoms.^10^ Specifically, the heterozygous c.620G>A (p.Arg207His) substitution, which has been recurrently identified in patients with an autosomal dominant *ATP5F1A*‐linked syndrome (Table S1), was demonstrated to lead to decreased ATP5F1A subunit protein levels on immunoblot studies,^10^ a result comparable with the outcome of our evaluation shown in Figure 3. The authors of this prior study concluded that it was unclear whether the disease‐causal nature of the *ATP5F1A* c.620G>A (p.Arg207His) variant resulted from an LoF or from a dominant‐negative effect.^10^ Based on the data presented in this work, we would argue that the loss of ATP5F1A expression observed in association with *ATP5F1A* c.620G>A (p.Arg207His)^10^ and P1's frameshift alteration is probably linked to the phenotypic abnormalities, and that this should be further investigated in suitable in vivo model systems. Despite the evidence provided, we acknowledge that there is currently no experimental data that would clearly establish the inability of a single functional copy of *ATP5F1A* to prevent the manifestation of disease. This would need to be proven in future functional work that implements methodology for accurate demonstration of individual ATP5F1A dose‐dependent effects on phenotypic outcomes. Moreover, a shortcoming of the characterization of our individual P1 is that we were unable to provide insights into familial segregation of the *ATP5F1A* variant because the parents were deceased and other relatives were unavailable for genetic analysis. Extended family segregation studies should be done in follow‐up assessments of other individuals with rare *ATP5F1A* variants, recruited via collaborations globally.

Furthermore, we identified a new recurrent missense mutation underlying dominant *ATP5F1A*‐related disease. By studying patients with this variant (individuals P2–P4), we determined that their clinical manifestations were less complicated than those of previously described patients^7^, ^10^ and more similar to typical forms of HSP, such as SPG3A or SPG4.^34^ Despite the lack of functional data, pathogenicity of the missense variant is very likely given its predicted deleteriousness, absence from controls, familial segregation/de novo mutagenesis, and recurrence in affected subjects; interestingly, the same variant is listed as likely pathogenic/pathogenic in two additional ClinVar entries without phenotype availability (accessed September 2024, variation ID: 2572074).^35^ It remains to be determined whether this particular missense variant acts as an LoF or dominant‐negative allele. Dominant‐negative effects have been discussed for *ATP5F1A* missense variants located on α‐/β‐subunit interaction surfaces of ATPase.^7^, ^9^

Finally, this study offers the first report of an *ATP5F1B* splice‐site mutation in a patient with infantile‐onset dystonic features. The two reported monoallelic dystonia‐associated *ATP5F1B* variants resulted in substitutions of single amino acid residues,^9^ and no other predicted LoF alleles in *ATP5F1B* have been clinically documented as pathogenic until today. Individual P5 was diagnosed with CP, in contrast with recently published patients with isolated dystonia.^9^ The observed loss of correctly spliced exons with accompanying significant reduction in *ATP5F1B* mRNA levels and the activity defect of ATPase in P5's cells were strongly suggestive of a disease‐causing character of the variant. Moreover, the finding that both the *ATP5F1B* splice‐site variant (individual P5) and the *ATP5F1A* frameshift variant (individual P1) compromised the enzymatic activity of ATPase in a similar fashion (Fig. 4) may suggest that the mutations could lead to functionally similar pathological outcomes. Nevertheless, we emphasize that additional experimental studies are warranted to further dissect the mechanism of action of *ATP5F1B* variants; because a cell line of P5's mother, who also carried the splice‐site variant, was not available, we could not perform a comparison of the relative levels of *ATP5F1B* mRNA, ATPase protein subunits, and ATPase activity between this subject and her affected daughter. Lack of functional workup for the variant‐harboring mother represents another limitation of this study.

The mechanisms that cause movement disorders and neurodevelopmental abnormalities in dominantly inherited defects of ATPase have not yet been established. In addition to bioenergetic impairments, defective ATPase function can lead to increased generation of reactive oxygen species (ROS), by‐products of oxidative metabolism with diverse modulatory roles in neurodevelopment.^8^ Thus, aberrant ROS regulation may alter the control of redox‐sensitive molecules such as transcription factors and signaling effectors, including those expressed in dopaminergic neurons.^8^ Our observations underscore the importance of anticipating a broad phenotypic spectrum for such potential molecular alterations in *ATP5F1A*/*ATP5F1B*‐related conditions, ranging from isolated or complex dystonia to HSP and CP.

*ATP5F1A*/*ATP5F1B*‐related diseases display reduced penetrance.^9^ Like relatives in two published dystonia‐affected families,^9^ the mother of P5 carried a disease‐associated *ATP5F1B* variant but was asymptomatic. Moreover, several control individuals with putative *ATP5F1A*/*ATP5F1B* LoF alleles exist in the newest gnomAD v4.1.0 version (https://gnomad.broadinstitute.org/). Based on metrics from gnomAD v2.1.1, for which still most experience exists in the use for gene and variant prioritization, *ATP5F1A* and *ATP5F1B* are severely constrained against LoF variants (probability of being LoF intolerant [pLI] scores of 1.0 and 0.98, respectively; LOEUF scores of 0.18 and 0.31, respectively). Notably, it is an increasingly recognized phenomenon that LoF constraints for individual genes can be less pronounced in gnomAD v4.1.0 compared with v2.1.1, especially in the context of hereditary movement disorders that are associated with reduced penetrance, because of inclusion of larger fractions of individuals from clinically ascertained cohorts and disease biobanks.^36^ Altogether, data from both gnomAD versions are, with robust statistical significance (pLI and/or LOEUF), clearly in favor of LoF intolerance of *ATP5F1A* and *ATP5F1B*.^6^ The explanations for potential nonmanifestation of disease in heterozygous carriers of *ATP5F1A*/*ATP5F1B* LoF variants still need to be elucidated, particularly in the context of different metabolic scenarios and other intrinsic or extrinsic influences. It is possible that environmental factors or other adverse secondary effects may affect the penetrance of *ATP5F1A*/*ATP5F1B*‐related conditions by further increasing energy demands and/or ROS production.^8^ Although fully speculative, we mention that such a trigger could have been the complicated birth of P5, similarly to what has been postulated for other CP‐affected subjects with combined mitochondrial and perinatal injuries.^37^, ^38^ Evidence from this and prior work^7^, ^9^, ^10^, ^12^, ^13^ suggests that the disease mechanism for heterozygous variants in *ATP5F1A*/*ATP5F1B* could be insufficient protein production (LoF/missense alleles) and dominant negative (missense alleles) and, for biallelic variants, (near‐)complete loss of protein function. Interestingly, there are several additional one‐gene, two‐mechanism examples of monogenic mitochondrial disorders. For example, *SLC25A4* variants are known to underlie a remarkable spectrum of clinically distinct mitochondrial defect syndromes that can be associated with dominantly transmitted, de novo, and biallelic recessive variants, operating via different mechanisms of pathogenicity.^39^ In clinical practice, we advise the implementation of clinicogenetic correlation and segregation analyses, the assessment of functional assay outcomes (eg, enzymatic measurements) whenever possible, as well as the application of sequence interpretation standards to evaluate the significance of an identified rare variant in *ATP5F1A*/*ATP5F1B*.^40^

Overall, this study confirms heterozygous variants in *ATP5F1A* and *ATP5F1B* as an underreported cause of a wider range of neurological phenotypes with prominent movement disorders and permits the determination of an expanded mutational spectrum. We highlight the need for future investigations of independent cohorts and functional outcomes to better define the impact and penetrance of different heterozygous variant types in these genes. In light of emerging therapeutic drug‐repurposing strategies targeting ATPase‐related encephalopathies,^41^ it will be crucial to further understand downstream mechanisms and the role of LoF and dominant‐negative variants in these disorders.

## Author Roles

Philip Harrer: study design and concept, acquisition of data, analysis and interpretation of data, and revision of manuscript for critical intellectual content. Magdalena Krygier: acquisition of data, analysis and interpretation of data, and revision of manuscript for critical intellectual content. Martin Krenn: acquisition of data, analysis and interpretation of data, and revision of manuscript for critical intellectual content. Volker Kittke: acquisition of data and revision of manuscript for critical intellectual content. Alice Saparov: acquisition of data and revision of manuscript for critical intellectual content. Virginie Pichon: acquisition of data and revision of manuscript for critical intellectual content. Marlène Malbos: acquisition of data and revision of manuscript for critical intellectual content. Clarisse Scherer: acquisition of data and revision of manuscript for critical intellectual content. Ivana Dzinovic: acquisition of data and revision of manuscript for critical intellectual content. Matej Skorvanek: acquisition of data and revision of manuscript for critical intellectual content. Robert Kopajtich: acquisition of data and revision of manuscript for critical intellectual content. Holger Prokisch: acquisition of data and revision of manuscript for critical intellectual content. Sara Silvaieh: acquisition of data and revision of manuscript for critical intellectual content. Anna Grisold: acquisition of data and revision of manuscript for critical intellectual content. Maria Mazurkiewicz‐Bełdzińska: acquisition of data and revision of manuscript for critical intellectual content. Jean‐Madeleine de Sainte Agathe: acquisition of data and revision of manuscript for critical intellectual content. Juliane Winkelmann: study supervision, and analysis and interpretation of data. Jan Necpal: study supervision, and analysis and interpretation of data. Robert Jech: study supervision, and analysis and interpretation of data. Michael Zech: study design and concept, study supervision, analysis and interpretation of data, and wrote the manuscript. Martin Danis: acquisition of data and revision of manuscript for critical intellectual content. Georgi Krastev: acquisition of data and revision of manuscript for critical intellectual content.

## Financial Disclosures (for the preceding 12 months)

The authors report no disclosures.

## Supporting information


**Data S1.** Supporting Information.

## Data Availability

The data that support the findings of this study are available on request from the corresponding author. The data are not publicly available due to privacy or ethical restrictions.
