# Supplementary material for: Expanding the Allelic and Clinical Heterogeneity of Movement Disorders Linked to Defects of Mitochondrial Adenosine Triphosphate Synthase
Source: Mov Disord. 2025 Apr 25;40(7):1388–400. doi: 10.1002/mds.30209 (PMC12273622; doi:10.1002/mds.30209)
Supplement: Supplementary file 1 — Data S1. Supporting Information. [file MDS-40-1388-s001.pdf]

Suppl. Table 1 Individuals with variants in the ATPase subunit genes *ATP5F1A* and *ATP5F1B* present with movement disorders and neurodevelopmental deficits

Family, Patient(s)	Family A	Family B	Family C		Family D	Zech et al.[1]	Zech et al.[1]	Zech et al.[1]	Lines et al.[2]	Nasca et al.[3]	Nasca et al.[3]
	P1	P2	P3	P4	P5	P5	P6	P7	P1-3	Family A: A-II-2, A-II-3, A-III-2	Family B: B-III-1
Sex, age	M, 43y	F, 22y	F, 32y	F, 61y	F, 14y	F, 14y	M, 17y	F, 12y	M, 3y; F, 14m; F, 18m	F, 48y; F, 47y; M, 20y	F, 27y
Geographic origin	Slovakia	Austria	France	France	Ukraine	Eu	Eu	NA	NA	Eu	Eu
Genomic testing	WGS	WES	WES	WES	WGS	WES	WES	WES	WES	WES	WES
**Variant details**											
Gene	*ATP5F1A*	*ATP5F1A*	*ATP5F1A*	*ATP5F1A*	*ATP5F1B*	*ATP5F1A*	*ATP5F1A*	*ATP5F1A*	*ATP5F1A*	*ATP5F1B*	*ATP5F1B*
cDNA variant	NM_004046.6: c.1404del	NM_004046.6: c.1252G>A	NM_004046.6: c.1252G>A	NM_004046.6: c.1252G>A	NM_001686.4: c.1074+1G>T	NM_004046.6: c.620G>A	NM_004046.6: c.545G>A	NM_004046.6: c.1037C>T	NM_004046.6: c.620G>A	NM_001686.4: c.1000A>C	NM_001686.4: c.1445T>C
Protein variant	NP_004037.1: p.Glu469Serfs*3	NP_004037.1: p.Gly418Arg	NP_004037.1: p.Gly418Arg	NP_004037.1: p.Gly418Arg	NP_001677.2: p.?	NP_004037.1: p.Arg207His	NP_004037.1: p.Arg182Gln	NP_004037.1: p.Ser346Phe	NP_004037.1: p.Arg207His	NP_001677.2: p.Thr334Pro	NP_001677.2: p.Val482Ala
In-silico prediction(s)	NA		CADD v1.6: 32 REVEL: 0.954 AlphaMissense: 0.9999		CADD v1.6: 36 SpliceAI: 1.0	NA[1]	NA[1]	NA[1]	NA[1]	NA[1]	NA[1]
gnomAD v4.1.0, in-house controls	not found	not found	not found	not found	not found	1/1614184, not found	not found	not found	1/1614184, not found	not found	1/1614130, not found
Zygosity	heterozygous	heterozygous	heterozygous	heterozygous	heterozygous	heterozygous	heterozygous	heterozygous	heterozygous	heterozygous	heterozygous
Inheritance	unknown	*de novo*	maternal	unknown	maternal	*de novo*	*de novo*	*de novo*	*de novo*	maternal	paternal
ACMG	likely pathogenic (PS3, PM2, PP3)		likely pathogenic (PS4_moderate, PM1, PM2, PM6, PP3)		likely pathogenic (PS3, PM2, PM4, PP3)	NA[1]	NA[1]	NA[1]	NA[1]	NA[1]	NA[1]
**Dystonia and other movement-disorder features**											
Dystonia	+ (childhood-onset generalized dystonia)	-	-	-	+ (infantile-onset generalized dystonia, dystonic CP)	-	+	+ (dystonic CP)	-	+ (childhood/adolescence-onset generalized isolated dystonia)	+ (childhood-onset generalized isolated dystonia)
Myoclonus	+	-	-	-	-	-	-	-	-	-	-

Spasticity/ pyramidal signs	+	+ (spastic paraplegia)	+ (spastic paraplegia)	+ (spastic paraplegia)	-	-	+	+	-	-	-
**Neurodevelopmental and other comorbidities**											
Developmental delay	+ (motor and cognitive)	+ (motor)	+ (speech)	-	+ (motor)	(+) (spontaneous resolution)	+	+	-	-	-
Hypotonia	+ (during infancy)	-	-	-	+	(+) (spontaneous resolution)	+	+	(+) (spontaneous resolution)	-	-
Intellectual impairment	+ (mild-moderate)	+ (mild)	+ (mild)	-	-	-	+	+	-	-	-
Seizures	-	-	-	-	-	-	-	-	-	-	-
Other features	-	+ (optic glioma)	autism spectrum disorder	-	-	failure-to-thrive	hearing impairment	swallowing problems; strabismus; 21-hydroxylase deficiency	failure-to-thrive, diarrhea, anaemia, primarily metabolic abnormalities (spontaneous resolution)	-	-
Brain MRI abnormality	-	- (except for optic glioma)	-	-	-	-	-	signal abnormality in the bilateral ventrolateral thalami	NA	-	slight asymmetry of lateral ventricles

Abbreviations: ACMG, American College of Medical Genetics and Genomics; CADD, Combined Annotation Dependent Depletion; CP, cerebral palsy; Eu, European; F, female; gnomAD, The Genome Aggregraion Database; M, male; m, months; minus (-) feature absent or not reported; MRI, magnetic resonance imaging; NA, not available/not applicable; plus (+), feature present; REVEL, Rare Exome Variant Ensemble Learner; WES, whole-exome sequencing; WGS, whole-genome sequencing; y, years.

[1](Re)evaluation of *in-silico* predictions and applied ACMG criteria for previously published cases not provided.

1. Zech M, Kopajtich R, Steinbrucker K, et al. Variants in Mitochondrial ATP Synthase Cause Variable Neurologic Phenotypes. Ann Neurol 2022;91(2):225-237.
2. Lines MA, Cuillerier A, Chakraborty P, et al. A recurrent de novo ATP5F1A substitution associated with neonatal complex V deficiency. Eur J Hum Genet 2021;29(11):1719-1724.
3. Nasca A, Mencacci NE, Invernizzi F, et al. Variants in ATP5F1B are associated with dominantly inherited dystonia. Brain 2023;146(7):2730-2738.

Suppl. Figure 1 Proteomics sample rank plots for expression of six ATPase protein subunits in patient P1 (family A)

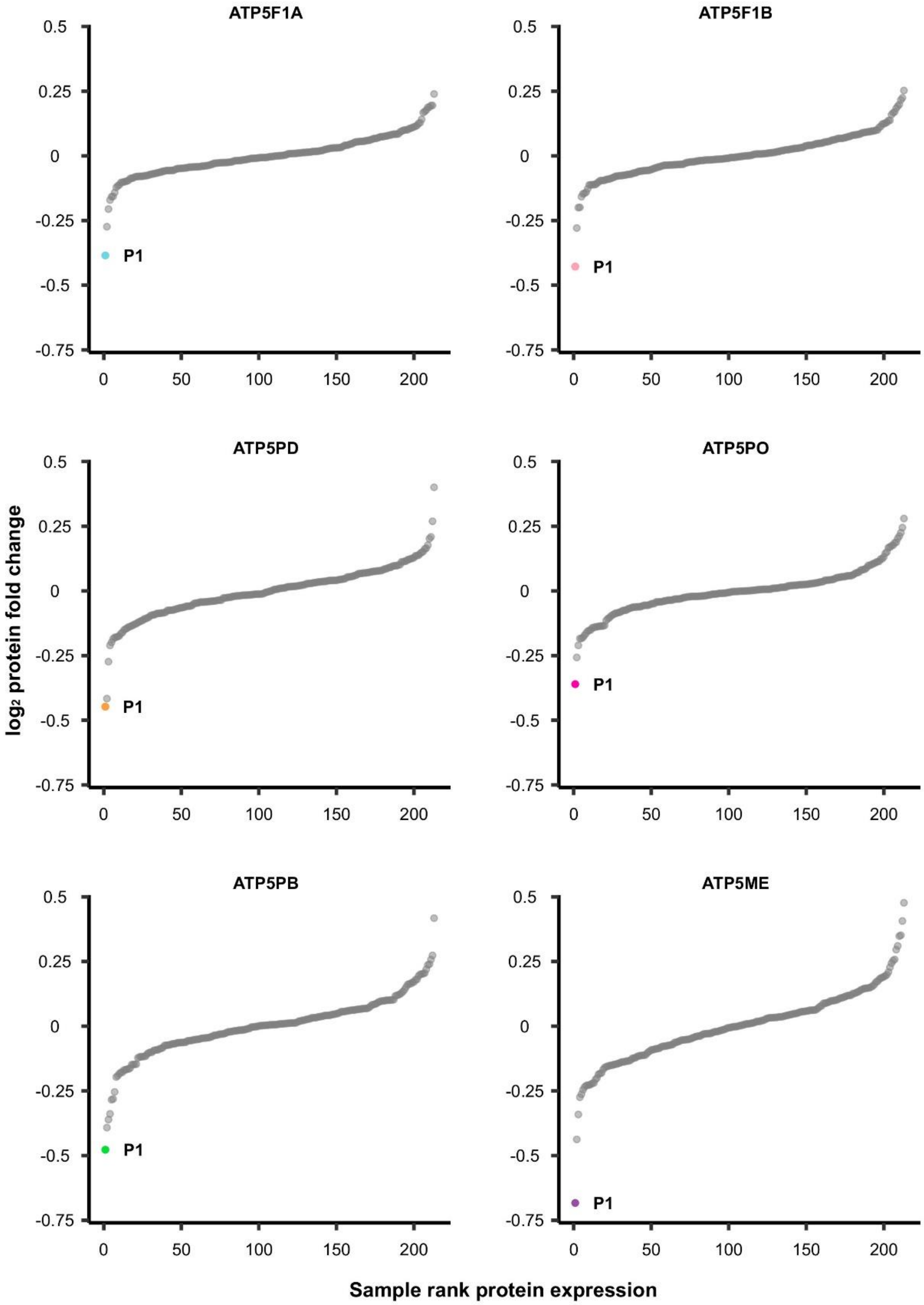

In comparison to a set of 212 available in-house proteome samples, P1 had the lowest amounts of ATP5F1A, ATP5F1B, ATP5PD, ATP5PO, ATP5PB, and ATP5ME (colored data points in the sample rank plots).
